# Mismatch Negativity and Cognitive Performance for the Prediction of Psychosis in Subjects with At-Risk Mental State

**DOI:** 10.1371/journal.pone.0054080

**Published:** 2013-01-17

**Authors:** Yuko Higuchi, Tomiki Sumiyoshi, Tomonori Seo, Tomohiro Miyanishi, Yasuhiro Kawasaki, Michio Suzuki

**Affiliations:** 1 Department of Neuropsychiatry, University of Toyama Graduate School of Medicine and Pharmaceutical Science, Toyama, Japan; 2 Department of Neuropsychiatry, Kanazawa Medical University, Uchinada-machi, Japan; RIKEN Brain Science Institution, Japan

## Abstract

**Background:**

A shorter duration of untreated psychosis has been associated with better prognosis in schizophrenia. In this study, we measured the duration mismatch negativity (dMMN), an event-related potential, and cognitive performance in subjects with at-risk mental state (ARMS), patients with first-episode or chronic schizophrenia, and healthy volunteers. The main interest was to determine if these neurocognitive measures predict progression to overt schizophrenia in ARMS subjects.

**Methodology/Principal Findings:**

Seventeen ARMS subjects, meeting the criteria of the Comprehensive Assessment of At-Risk Mental State, 31 schizophrenia patients (20 first-episode and 11 chronic) and healthy controls (N = 20) participated in the study. dMMN was measured by an auditory odd-ball paradigm at baseline. Neuropsychological performance was evaluated by the Japanese version of the Brief assessment of cognitive function of schizophrenia (BACS-J). The first-episode schizophrenia group showed significantly smaller amplitudes at frontal electrodes than did control subjects whereas chronic patients elicited smaller amplitudes at frontal and central electrodes, consistent with previous reports. During the follow-up period, 4 out of the 17 ARMS subjects transitioned to schizophrenia (converters) while 13 did not (non-converters). Specifically, dMMN amplitudes of non-converters did not differ from those of healthy controls, while converters showed significantly smaller dMMN amplitudes at some electrodes compared to control subjects. Converters performed significantly worse on tests of working memory, verbal fluency, and attention/information processing than did non-converters. There was a significant positive correlation between dMMN amplitudes at the frontal electrodes and verbal fluency, as measured by the BACS, in the AMRS subjects as a whole.

**Conclusions/Significance:**

ARMS subjects who later developed schizophrenia elicited smaller dMMN amplitudes to begin with, compared to non-converters. Notably, we have provided the first evidence for the ability of verbal fluency to predict dMMN amplitudes in ARMS subjects. These findings are expected to add to the efforts for early diagnosis and intervention of schizophrenia.

## Introduction

Schizophrenia usually develops around the adolescence period, with the whole life risk of about 0.85% [1]. Patients with schizophrenia suffer from positive symptoms (hallucination, delusion, thought disturbance, and etc.), negative symptoms (blunted affect, lack of volition, social withdrawal and etc.), and impairment in a range of cognitive domains, e.g. several types of memory, executive function, attention, verbal fluency [2,3,4,5]. Especially, cognitive function is considered to be a major determinant of outcome, including quality of life and social function [6]. It is interesting that the classification of cognitive domains differs across neuropsychological test batteries. For example, verbal fluency is categorized as an independent domain in the Brief Assessment of Cognition in Schizophrenia (BACS) [7,8], while it is regarded as one of the components of processing speed (of information) in the Measurement and Treatment Research to Improve Cognition in Schizophrenia – Consensus Cognitive Battery [9].

In order to achieve satisfactory long-term outcome, early detection, intervention and treatment of schizophrenia are needed. Specifically, a shorter duration of untreated psychosis (DUP) has been associated with a greater response to antipsychotic drugs in terms of symptoms and quality of life [10]. Prolonged DUP is also associated with decreased levels of social functions, for example, work function and communication skills, as well as longer hospitalization [11], [12], [13], [14], [15], [16], [17]. In this context, it was reasonable that recent efforts have been directed to subjects with “at-risk mental state (ARMS)” or “ultra-high risk patients.” [18].

For the purpose of early diagnosis, objective biomarkers, particularly, those based on brain morphology, neurophysiology, and neuropsychology have been reported to provide useful information [19], [20], [21], [22], [23], [24]. Neurophysiological measurements, such as event-related potentials (ERPs), have been suggested to provide a biological substrate for some aspects of cognitive disturbances of schizophrenia. Especially, P300, mismatch negativity (MMN), or N400 etc. are widely used ERPs for this purpose. For example, schizophrenia patients show smaller amplitudes of P300 than normal control subjects [25], [26], [27]. Reduction of P300 amplitudes has been also noted in subjects with ARMS, part of which develops schizophrenia [28]. P300 has been shown to be affected by various factors, including medication [20], [29], [30] suggesting the utility as a state marker of psychotic disorders.

MMN is another component of ERPs generated in response to occasional variations (e.g., duration, frequency, intensity) of acoustic stimuli, and is suggested to reflect pre-attentive cognitive operations [31], [32]. MMN amplitudes have been shown to be decreased in patients with schizophrenia, as indicated by a recent meta-analysis [33] reporting a large effect size. Unlike the case with P300, MMN amplitudes are generally not affected by psychotropic drug, for example benzodiazepines [34], dopamine antagonists [35]. For these reasons, MMN is considered to provide a trait marker for schizophrenia.

There are several types of MMNs, such as duration MMN (dMMN) and frequency MMN (fMMN), based on the mode of presentation of stimuli. Attenuation of the fMMN amplitude, resulting from changes in the frequency of stimuli, reflects the progress of the disease, i.e. a function of duration of the illness. On the other hand, deficits of dMMN deficiency, resulting from changes in the duration of stimuli, may be more closely linked to the genetic aspect of schizophrenia [36]. Thus, impairment of dMMN is greater than that of fMMN [37], with the latter emerging only in the chronic, but not early stage of schizophrenia [38], [39].

Recently, dMMN amplitudes have been shown to be reduced already in the prodromal stage of schizophrenia. Thus, Jahshan et al (2011) found dMMN amplitudes in subjects with at-risk for psychosis patients were reduced compared to normal controls, but the deficits were milder than those in patients with first episode schizophrenia [40]. Atkinson et al (2011) report that dMMN amplitudes were reduced as early as in the ultra-high risk stage [41]. This finding was extended by Bodatsch et al (2011) [42] and Shaikh et al (2012) [43], who observed smaller dMMN amplitudes in drug-naïve subjects with ARMS who later converted to overt psychosis, compared to those in non-converters. Thus, reduced dMMN amplitudes have been regarded to provide a biomarker to predict the development of schizophrenia.

Cognitive impairment, a core symptom of schizophrenia, is present at onset of illness [44], and is closely related to functional outcome [45]. Carrion et al. (2011) observed that cognitive and functional impairments are already evident in ultra-high risk patients before the onset of psychosis. Specifically, attention/processing speed was found to predict progression to psychosis [46]. On the other hand, Frommann et al. (2011) report prodromal patients were impaired in all neurocognitive domains, such as learning memory, executive control, processing speed, and working memory. These findings indicate neuropsychological measures, particularly attention/processing speed, provide another cognitive modality to identify high-risk people vulnerable to developing overt schizophrenia [47].

To date, little information is available about the relationship between neurophysiological indices, e.g. dMMN, and neuropsychological performance. So far, Lin et al (2012) investigated the correlation between neuropsychological performance and MMN amplitudes only in patients with schizophrenia [21]. For example, demonstration of the ability of some measures of neuropsychological performance, e.g. attention/information processing and verbal fluency, to predict dMMN activity would greatly facilitate the early intervention practice, as the former indices require only a limited time constraint. Moreover, such evidence, if obtained, would help more precisely identify biological features of the prodromal phase of schizophrenia.

In this study, we measured dMMN amplitudes and cognitive performance in subjects with ARMS, first episode schizophrenia, or chronic phase of the illness, and compared them with those of normal control subjects. Specifically, we compared the results from ARMS subjects who later developed schizophrenia (converters) and those who did not (non-converters). The hypotheses tested were; 1) if correlations exist between the decrease in dMMN amplitudes and the impairment of neuropsychological performance in subjects with ARMS, and 2) if the impairments of neurophysiological and neuropsychological functions would similarly predict progression to overt psychosis in these subjects.

## Methods

### Ethics Statement

This protocol was approved by the Committee on Medical Ethics of the University of Toyama. After complete and detail description of the study to the subjects, written informed consent was obtained.

Clinical staff explained the nature of the study to the subjects, the risks and benefits, and the option not to participate in research. If the mental status of a subject was impaired to the point where s/he could not understand these issues, the subject was not approached to be in the research. In case there was a possibility that the capacity of a participant to consent was compromised, an additional consent was obtained from next of kin, care takers, or guardians of such subject.

### Participants

Diagnosis was made based on the Structured Clinical Interview for DSM-IV (SCID) for schizophrenia and the Comprehensive Assessment of At-Risk Mental State (CAARMS) for ARMS [48], by experienced psychiatrists. Most of these subjects were referred from “Psychiatric Health and Welfare Center of Toyama (PHWCT). Seventeen ARMS subjects followed at the University of Toyama Hospital participated in this study. [male/female = 4/13; mean (S.D.) age = 19.4(4.4)]. Thirty-one schizophrenia patients also participated in this study. Patients with duration of illness less than two years were defined as first episode schizophrenia (FES) [n = 20; male/female = 9/11; mean (S.D.) age = 27.2(7.3)], while those with duration of illness 2 years or longer were defined as chronic schizophrenia (CS) [n = 11; male/female = 6/5; mean (S.D.) age = 28.1(8.0)]. We recruited normal control subjects from the community by advertisements. They are healthy volunteers [n = 20; male/female = 14/6; mean (S.D.) age = 25.4(6.9)] without any personal history of psychiatric illnesses, including schizophrenia or other psychotic disorders.

All participants were right-handed. A psychiatric and treatment history was obtained from the subjects, families, and medical records. Subjects with a current history of substance abuse or dependence, seizure or head injury were excluded from the study. Eligible patients had a complete physical examination and standard laboratory testing was normal. Demographic data at baseline evaluation are shown in Table 1.

**Table 1:** Demographic and clinical data and dMMN amplitude.

	Healthy controls(n = 20)	ARMS(n = 17)	First episode schizophrenia(n = 20)	Chronic schizophrenia (n = 11)
Male/female	14/6	4/13	9/11	6/5
Age (years)	25.4 (6.9), range 16–45	19.4 (4.4)*, range, 15–29	27.2 (7.3), range 16–38	28.1 (8.0), range 18–44
Age of onset (years)	–	–	26.5 (7.1)	20.2 (4.7)
Duration of illness (years)	–	–	0.65 (0.51)	7.9 (6.9)
Drug dose a)	–	0.1(0.4)	2.1(2.3)	3.2 (2.4)
SAPS	–	13.2 (9.3)	15.7 (13.1)	17.6 (19.1)
SANS	–	50.3 (20.1)	53.8 (25.9)	51.5 (26.1)
dMMN amplitude[µV]				
F3	7.5 (1.3)	7.6 (2.2)	5.3 (1.5) **	4.5 (1.0) **
F4	7.3 (1.2)	7.5 (2.1)	5.6 (1.8) *	5.0 (1.3) **
Fz	7.9 (1.1)	7.9 (2.1)	5.6 (1.7) **	5.1 (1.7) **
Cz	6.6 (1.5)	6.6 (2.2)	5.1 (1.5)	4.2 (1.7) **
Pz	4.5 (1.7)	4.2 (2.0)	3.5 (1.2)	2.5 (1.0) **

Values represent mean (SD).

a) Risperidone equivalent [mg/day].

ARMS, at-risk mental state.

SAPS, Scale for the Assessment of Positive Symptoms;

SANS, Scale for the Assessment of Negative Symptoms.

* p<0.05 and ** p<0.01, compared to healthy control.

ARMS subjects were followed-up continuously at the hospital. Four out of the 17 ARMS subjects transitioned to schizophrenia during the observation period. When DSM-IV criteria were met, e.g. auditory hallucinations persisted or any delusion (for example, disturbance of the self) clearly observed, the subject was regarded to have converted to schizophrenia (converters; Conv.). Subjects who did not develop psychosis were defined as non-converters (Non-C.). The average observation period for ARMS subjects was 2.1±1.1 (Non-C.; 1.6±0.8) years.

### Clinical Assessment

The Scale for the Assessment of Positive Symptoms (SAPS) and the Scale for the Assessment of Negative Symptoms (SANS) [49] were administered by an experienced psychiatrist. These data are shown in Table 1.

### Neuropsychological Assessments

Neuropsychological performance, measured by the Japanese version of the BACS (BACS-J) [8], was evaluated by experienced psychiatrists or psychologists. The BACS-J cognitive battery uses the following assessments in the respective targeted domains: list learning (verbal memory), digit sequencing task (working memory), token motor task (motor function), category fluency and letter fluency (verbal fluency), symbol coding (attention and processing speed), and the Tower of London test (executive function), as shown in Table 1.

### Electroencephalogram Recording

Electroencephalograms (EEGs) were recorded based on the previous report of our laboratory [20], [30], [50], [51], [52], [53]. A 32-channel DC-amplifier (EEG-2100 version 2.22J,Nihon Kohden Corp., Tokyo, Japan), according to the international 10–20 system was used, and recordings were performed using an electro cap (Electrocap Inc., Eaton, OH) in a sound-attenuated room. Data were collected with a sampling rate of 500 Hz. All electrodes were referred to the average amplitude of the ear electrodes (bandwidth = 0.53–120 Hz, 60 Hz notch filter). Electrode impedance was less than 5 kΩ.

Measurements of dMMN were based on our previous report [53]. One thousand auditory stimuli were delivered binaurally through headphones with inter-stimulus intervals 500 msec. Standard/target tones of 50/100 msec duration were randomly presented with the presentation probability of 0.9/0.1. All tones were 60 dB, 1000 Hz and with a rise-fall time of 10 msec. The subjects were requested to watch silent animation movie (Tom and Jerry) and pay attention to the monitor and ignore the tones.

Averaging of ERP waves and related procedures were performed using Vital Tracer and EPLYZER II software (Kissei Comtec, Co. Ltd. Nagano, Japan). Epochs were 600 msec, including a 100-msec pre-stimulus baseline. Eye movement artifacts (blinks and eye movements) were manually rejected. MMN waveforms were obtained by subtract standard waveforms from target ones. ERP component peaks were identified within the 150–250 msec search windows. We selected F3, F4, Fz, Cz and Pz electrodes for analysis, based on our previous report [49].

### Statistical Methods

Statistical analyses were performed using the Statistical Package for Social Sciences (SPSS) version 19.0 (SPSS Japan Inc., Tokyo, Japan). In order to investigate group differences in MMN, repeated measures analysis of variance (ANOVA) with electrode site as within-subject variable and diagnostic group as between-subject variable was performed. BACS-J domain scores were analyzed with a two-way ANOVA with BACS-J domains as the within factor and group as the between factor. Group×electrode interactions and group×BACS-J domain score interactions were decomposed using one-way ANOVA, with Bonferroni correction. Relationships between MMN amplitudes at the Fz electrode and BACS -J domain scores were analyzed using Spearman rank correlations.

Raters (psychiatrist, psychologist) were not informed of subjects’ profiles and diagnosis.

## Results

### Subjects’ Profile

Demographic data of participants are shown in Tables 1 and 2. There was significant group difference in age [F(3,64) = 5.51, p = 0.02]. The ARMS group was significantly younger than other groups. The female to male ratio in the ARMS group was significantly greater than that in the normal control group [χ^2^ = 7.94, p = 0.004]. There was no difference between Conv. and Non-C. in age (p = 0.14). The male/female ratio of Conv. was greater than Non-C.[χ^2^ = 4.41, p = 0.01]. Fourteen out of 17 ARMS subjects were not taking any medication, and 3 were prescribed a small dose of risperidone (1.5 mg/day), aripiprazole (6 mg/day), and sulpiride (150 mg/day), respectively, for (or to prevent) acute psychosis episodes (sometimes with strong agitation), based on the criteria of International Early Psychosis Association Writing Group [54]. MMN recordings for these subjects were conducted shortly after medications were started (9, 15 and 27 days). All of the three subjects subsequently developed schizophrenia. Schizophrenia patients were taking the following treatment; FES (no medication 7, risperidone 3, perospirone 3, aripiprazole 2, olanzapine 1, sulpiride 1, blonanserin+quetiapine 1, risperidone+quetiapine 1, risperidone+zotepine 1.), CS (no medication 1, perospirone 3, risperidone 2, olanzapine 2, zotepine 1, perospirone+olanzapine 1, perospirone+aripiprazole 1). There were no differences between ARMS, FES and CS groups in SAPS [F(2,47) = 0.457, p = 0.636] and SANS [F(2,47) = 0.118, p = 0.889] scores. Conv. and Non-C. groups did not differ in the SAPS score. However, Conv. group showed a significantly higher score of SANS than Non-C. group (69.0±18.4 vs. 42.9±15.9, p = 0.02).

**Table 2:** Comparison between converters and non-converters of ARMS subjects.

	ARMS (n = 17)		Analyze of variance (df = 1,16), Group Effect	
	Non-C. (n = 13)	Conv. (n = 4)	F	p
Male/female	2/11	2/2		
Age [years]	18.5 (3.8), range 15–29	22.3 (5.6), range 17–30		
Drug dose ^a)^	–	0.5 (0.7)		
SAPS	11.4 (9.3)	18.0 (8.6)		
SANS	42.9 (15.9)	69.0 (18.4) *		
dMMN amplitude[µV]				
F3	8.2 (2.0)	5.6 (1.7)	3.78	n.s.
F4	8.2 (1.6)	5.2 (1.8)	10.61	0.05
Fz	8.6 (1.6)	5.7 (2.0)	8.25	0.01
Cz	7.3 (1.8)	4.3 (1.7)	8.31	0.01
Pz	4.8 (1.8)	2.4 (1.2)	4.74	0.04
BACS-J				
Verbal memory	51.0 (7.8)	47.2 (11.3)	0.57	n.s.
Working memory	19.1 (3.2)	14.7 (2.2)	6.33	0.02
Motor function	69.3 (12.5)	60.5 (9.0)	1.66	n.s.
Verbal fluency	46.7 (12.1)	29.0 (9.5)	7.03	0.01
Attention	74.0 (12.7)	56.2 (5.8)	7.05	0.01
Executive function	17.8 (2.1)	18.5 (2.6)	0.24	n.s.

Values represent mean (SD).

a) Risperidone equivalent [mg/day].

ARMS, at-risk mental state.

Non-C., ARMS non-converters; Conv., ARMS converters.

SAPS, Scale for the Assessment of Positive Symptoms;

SANS, Scale for the Assessment of Negative Symptoms;

BACS-J, Brief Assessment of Cognition in Schizophrenia, Japanese version.

* p<0.05 compared to Non-C. (student’s t-test).

### Comparisons of dMMN Amplitudes between Healthy Controls vs. ARMS vs. Schizophrenia

**Figure 1:**
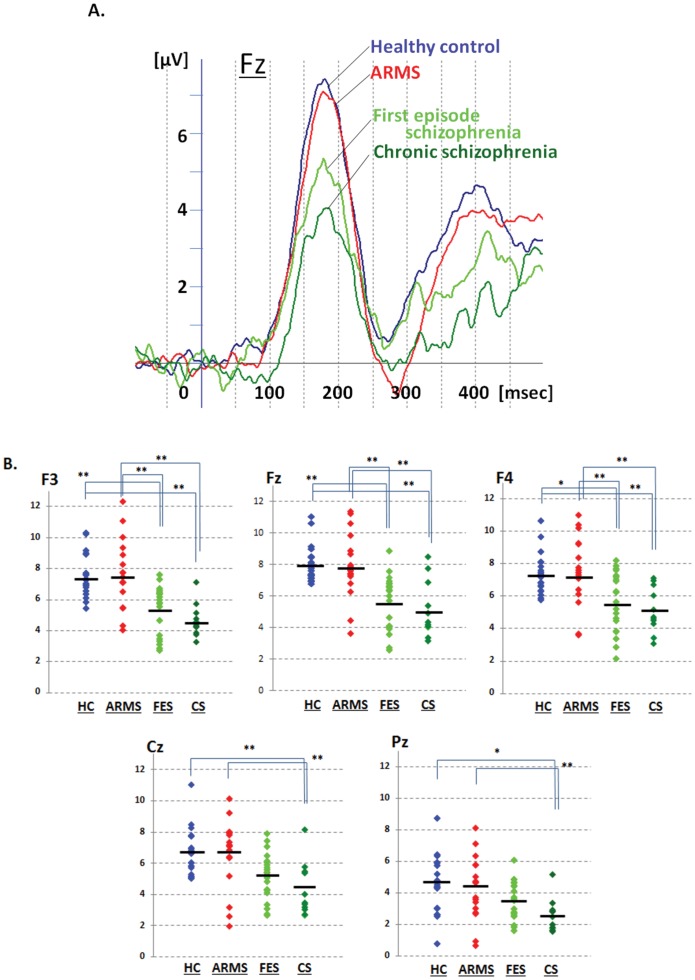
Duration mismatch negativity (dMMN) waveform at Fz and scatterplots of dMMN amplitudes for all subjects. A. Waveforms are presented for healthy controls (HC, blue line), at-risk mental state (ARMS, red line), first episode schizophrenia (FES, light green line) and chronic schizophrenia (CS, dark green line). B. Distribution of amplitudes are presented for healthy controls (HC, blue dots), ARMS (red dots), first episode schizophrenia (light green dots) and chronic schizophrenia (dark green dots). * p<0.05 and ** p<0.01, compared to each groups.

dMMN data are shown in Table 1 and Figure 1. Grand average waveforms in the Fz lead and scatterplots for the electrodes sites are shown in Figure 1A and 1B. ARMS subjects showed dMMN amplitudes similar to those of healthy control subjects. On the other hand, FES group showed significantly smaller dMMN amplitudes at frontal electrodes (F3, F4 and Fz). Patients with CS showed greater amplitude reductions at all electrodes compared to healthy controls.

### Comparisons of dMMN Amplitudes: Conv. vs. Non-C

**Figure 2:**
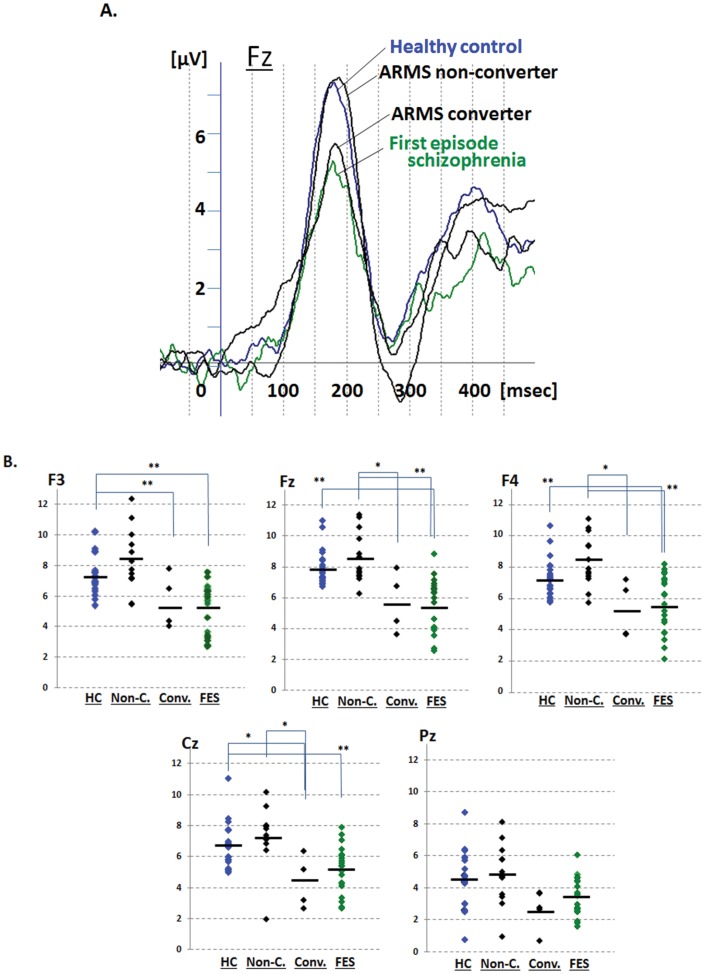
dMMN waveform at Fz and scatterplots of dMMN amplitude for at-risk mental state (ARMS,) healthy control (HC) and first episode schizophrenia (FES) subjects. A. Waveforms are presented for healthy controls (blue line), ARMS, converters (Conv.) and non-converter (Non-C.) (black lines), FES (light green line). B. Distribution of amplitudes are presented for healthy controls (blue dots), ARMS, converters (Conv.) and non-converter (Non-C.) (black dots), FES (light green dots). * p<0.05 and ** p<0.01, compared to each groups.

Conv. subjects showed significant reduction in dMMN amplitudes at F4, Fz, Cz, and Pz electrode sites compared with Non-C. subjects (Table 2, Figure 2A). Waveforms of Conv. were similar to those of first-episode schizophrenia. By contrast, waveforms of Non-C. resembled to those of healthy controls (Figure 2A). Scatterplots of dMMN amplitudes are shown in Figure 2B. Non-C. subjects elicited larger dMMN amplitudes compared to those of Conv. Amplitudes of Non-C. did not differ from those of healthy controls. On the other hand, Conv. showed significantly smaller dMMN amplitudes at F3 and Cz compared to control subjects. There were no differences in dMMN amplitudes at any electrode between Conv. and FES subjects.

### Neuropsychological Measurements: Conv. vs. Non-C

**Figure 3:**
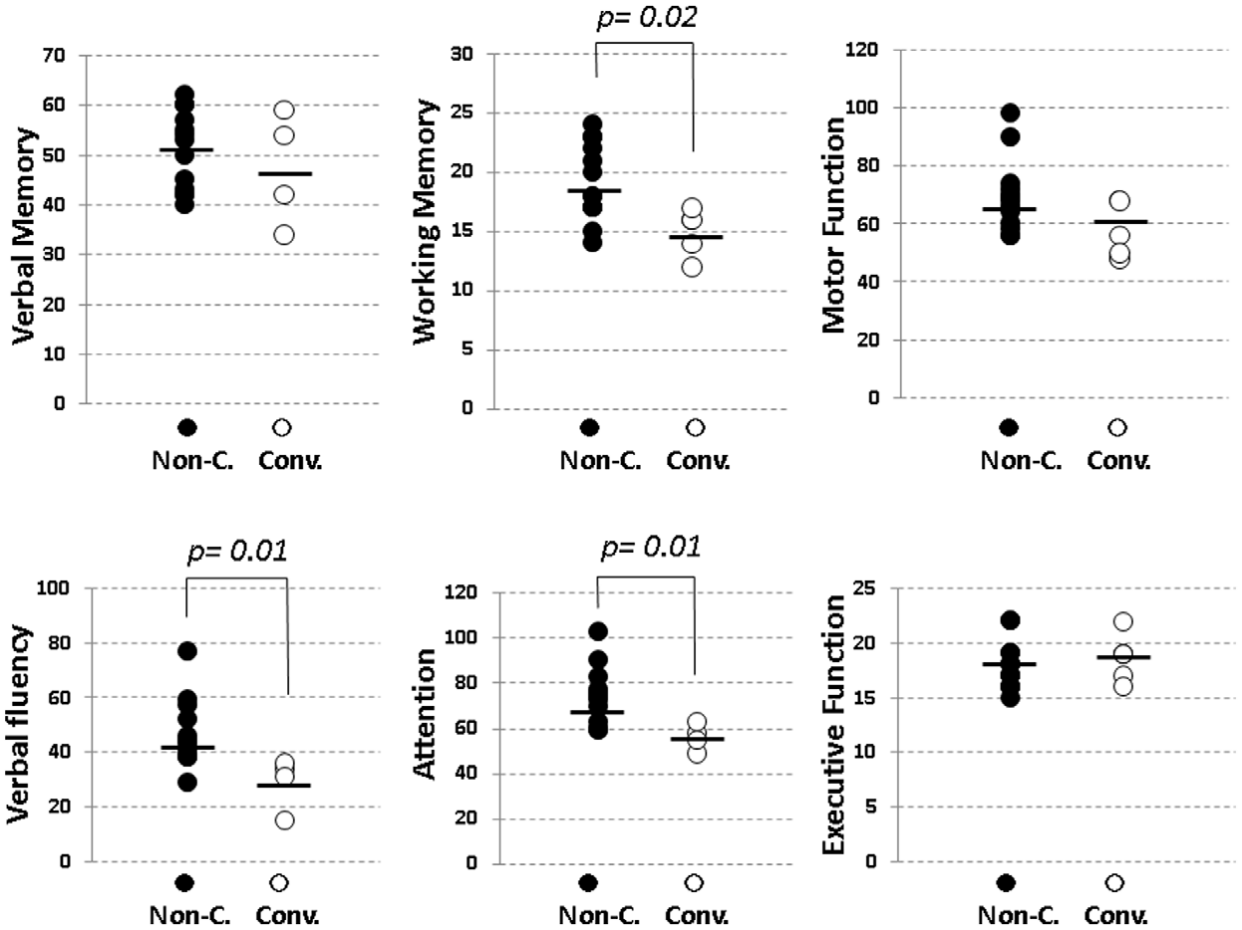
Scatterplot of the score of BACS-J for ARMS subjects. Black symbols(•) and white ones(○) represent scores of non-converters and converters, respectively.

Conv. subjects demonstrated significantly smaller BACS-J scores compared to Non-Conv. subjects for working memory, verbal fluency, and attention (Table 2, Figure 3).

### Relationship between Cognitive Performance and dMMN Amplitudes in ARMS subjects

**Figure 4:**
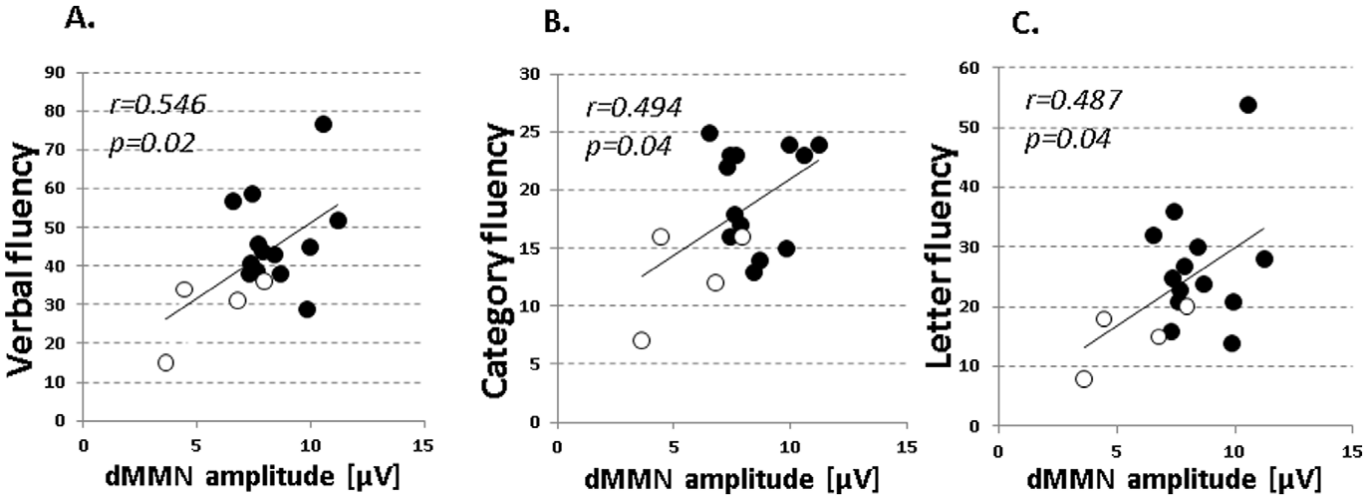
Correlations between dMMN amplitudes at Fz lead and performance on the verbal fluency tasks from the BACS-J in ARMS subjects. Black and white symbols represent scores of non-converters and converters, respectively. Relationships were analyzed using Pearson’s product-moment correlation coefficient.

Figure 4 demonstrates correlations between dMMN amplitudes and BACS scores in subjects with ARMS. Significant positive correlations were noted for verbal fluency (r = 0.546, p = 0.02; Figure 4A), but not other cognitive domains (data not shown). Also, scores of letter fluency task and category fluency task from the BACS-J [55] were significantly correlated with dMMN amplitudes in subjects with ARMS (Figure 4B,C).

## Discussion

To our knowledge, this study is the first to report a relationship between dMMN amplitudes and neuropsychological performance in individuals with ARMS. ARMS subjects who later converted to overt schizophrenia elicited reduced dMMN amplitudes at frontal and central leads compared with non-converters and normal subjects, consistent with previous reports [42], [43]. In addition, verbal fluency, working memory and attention/information processing were more greatly impaired in converters compared to non-converters at baseline. Further a significant correlation was noted between performance on verbal fluency tasks and dMMN amplitudes in ARMS subjects. First episode schizophrenia patients showed significantly smaller dMMN amplitudes than ARMS subjects and healthy controls, consistent with previous observations [40], [56]. Yung et al. (2003) [57] report that 10–40% of ARMS patients develop schizophrenia, consistent with our observations that 4 out of 17 (23.5%) subjects progressed to overt psychosis. Some previous studies report that ARMS subjects elicit reduced dMMN amplitudes, but with a lesser degree compared to patients with established schizophrenia [40], [41], [42]. By contrast, dMMN amplitudes of the entire ARMS subjects in the present study were not significantly different from those of healthy controls (Figure 1). One of the reasons for this discrepancy is the difference in age and the percentage of gender, as implicated by some previous studies [32], [58], [59].

The score of SANS/SAPS of ARMS were similar to schizophrenia (Table 1). We consider it was due, mainly, to the nature of the ARMS subjects studied here. Most of these subjects were referred from PHWCT. The PHWCT, a component of the Consultation and Support Service in Toyama(CAST), includes the Local Support Center for Social Withdrawal Young People that advertises its activity using internet home page and pamphlets. These systems mainly receive consultations from the family members of subjects with social withdrawal and/or disability. This may be why the ARMS subjects studied here elicited relatively severe negative symptoms comparable to those in subjects with overt schizophrenia. With regard to SAPS scores, part of the schizophrenia patients in this study had already been medicated, which may have decreased positive symptoms in these subjects. This may make the SAPS scores for ARMS group and schizophrenia groups look somewhat similar.

Compared to non-converters, dMMN amplitudes in converters were significantly reduced at F4, Fz, Cz and Pz leads (Table 2). This finding suggests dMMN amplitudes may be able to differentiate high-risk individuals who convert to schizophrenia from those who do not. Therefore, these electrophysiological findings are expected to facilitate early intervention of schizophrenia.

MMN is a pre-attentional response to a change of stimuli, and plays a critical role in establishing learning and memory. This electrophysiological event has been suggested to be generated by the glutamate (Glu)/N-methyl-D-aspartate (NMDA) system [60]. This theory is supported by the observation that administration of an NMDA-receptor antagonist (phencyclidine, MK-801 etc.) abolishes MMN in monkeys [61] and rats [62], [63]. The pathophysiology of schizophrenia has been shown to be associated with the dysfunction of signal transduction through NMDA receptors [64]. Accordingly, Stone et al. (2009) report that ARMS subjects elicited reduced Glu levels in the thalamus, which was correlated with the gray matter volume of frontal and temporal lobes [65], the brain structures suggested to be involved in MMN generation [66], [67]. In fact, the results of the present study (Table 2, Figure 2) indicate the ability of diminished dMMN to predict the development of schizophrenia, as in some previous reports [36]–[39], suggesting impaired NMDA-mediated transmissions provide an endophenotype for subjects vulnerable to the illness.

Neuropsychological deficits have been shown to exist in the early stage of schizophrenia [46], [47]. In this study, neuropsychological performance, as measured by the BACS, differentiated between converters and non-converters in ARMS subjects. Compared with non-converters, scores of working memory, verbal fluency and attention in converters were significantly less for converters (Table 2, Figure 3). These results indicate cognitive abilities, particularly those requiring attention/information processing speed, provides a sensitive marker predicting the development of schizophrenia in vulnerable individuals.

The major finding of the present study was the ability of performance on the verbal fluency tasks to predict dMMN amplitudes in subjects with ARMS (Figure 4). The implications of these observations include the possibility of enhancing accuracy to identify subjects diagnosed with “ultra-high risk” who later develop psychosis. Another advantage is that some neuropsychological tests, which only require a shorter time constraint, could substitute for electrophysiological measurements, e.g. ERPs. In fact, verbal fluency test only requires less than 5 minutes. The easiness of assessment would facilitate the screening for subjects whose psychiatric conditions would not allow them to undergo ERPs measurement, which generally takes more than 30 minutes. On the other hand, neuropsychological evaluations may sometimes be influenced by motivation of examinees. Therefore, combined administration of neurophysiological and neuropsychological assessments would facilitate screening procedures, depending on the condition of patients. In sum, these efforts are likely to lead to improvement of functional outcome for vulnerable subjects through early intervention by objective probes with greater sensitivity and specificity.

In conclusion, this study confirmed that ARMS subjects who later develop schizophrenia elicit smaller dMMN amplitudes to begin with, compared to non-converters. Notably, we have provided the first evidence for the ability of verbal fluency or attention/information processing to predict dMMN amplitudes in ARMS subjects. These findings are expected to add to the efforts for early diagnosis and intervention of schizophrenia.

### Limitations

The main limitations of this study include that ARMS subjects were younger and had a larger female/male ratio compared to other groups. Clearly, further study with a larger number of matched subjects is warranted. Part of ARMS subjects was taking antipsychotic drugs which is another limitation of the study.

The observation periods of Non-C. were relatively short (1.6±0.8 year), compared to similar studies [42], [43], which might be another limitation.
